# Identification of key biomarkers related to fibrocartilage chondrocytes for osteoarthritis based on bulk, single-cell transcriptomic data

**DOI:** 10.3389/fimmu.2024.1482361

**Published:** 2024-11-21

**Authors:** Bailin Pan, Peixiu Yao, Jinjin Ma, Xuanhao Lin, Laixi Zhou, Canzhen Lin, Yufeng Zhang, Bendan Lin, Chuangxin Lin

**Affiliations:** ^1^ Department of Orthopedics, Sun Yat-sen Memorial Hospital, Sun Yat-sen University, Guangzhou, Guangdong, China; ^2^ Department of Orthopedic Surgery, Shantou Central Hospital, Shantou, Guangdong, China; ^3^ Department of Biobank, Shantou Central Hospital, Shantou, Guangdong, China; ^4^ Institute of Future Health, South China University of Technology, Guangzhou, China; ^5^ Department of Graduate Student, Shantou University Medical College, Shantou, Guangdong, China

**Keywords:** Osteoarthritis, Cartilage, fibrocartilage chondrocytes, biomarkers, single-cell RNA sequencing, WGCNA, LASSO regression

## Abstract

**Introduction:**

Osteoarthritis (OA) is a prevalent joint disease that severely impacts patients’ quality of life. Due to its unclear pathogenesis and lack of effective therapeutic targets, discovering new biomarkers for OA is essential. Recently, the role of chondrocyte subpopulations in OA progression has gained significant attention, offering potential insights into the disease. This study aimed to explore the role of fibrocartilage chondrocytes (FC) in the progression of OA and identify key biomarkers related to FC.

**Methods:**

We analyzed single-cell ribonucleic acid sequencing (scRNA-seq) data from samples of OA and normal cartilage, focusing on FC. Microarray data were integrated to identify differentially expressed genes (DEGs). We conducted functional-enrichment analyses, including Kyoto Encyclopedia of Genes and Genomes (KEGG) and Gene Ontology (GO), and used weighted gene co-expression network analysis (WGCNA) and the least absolute shrinkage and selection operator (LASSO) algorithm to select biomarkers. A novel risk model for OA was constructed using these biomarkers. We then built a transcription factor (TF)–gene interaction network and performed immunohistochemistry (IHC) to validate protein expression levels of these biomarkers in cartilage samples.

**Results:**

The study identified 545 marker genes associated with FC in OA. GO and KEGG analyses revealed their biological functions; microarray analysis identified 243 DEGs on which functional-enrichment analysis were conducted. Using WGCNA and LASSO, we identified six hub genes, on the basis of which we constructed a risk model for OA. In addition, correlation analysis revealed a close association between Forkhead Box (FoxO)-mediated transcription and these these biomarkers. IHC showed significantly lower protein levels of ABCA5, ABCA6 and SLC7A8 in OA samples than in normal samples.

**Conclusion:**

This study used a multi-omics approach to identify six FC-related OA biomarkers (BCL6, ABCA5, ABCA6, CITED2, NR1D1, and SLC7A8) and developed an exploratory risk model. Functional enrichment analysis revealed that the FoxO pathway may be linked to these markers, particularly implicating ABCA5 and ABCA6 in cholesterol homeostasis within chondrocytes. These findings highlight ABCA family members as novel contributors to OA pathogenesis and suggest new therapeutic targets.

## 1 Introduction

Osteoarthritis (OA) is one of the most common chronic joint diseases, significantly affecting patients’ quality of life and imposing substantial socioeconomic burdens. The major clinical symptoms of OA include joint pain, limited mobility, and eventual disability (1). As the population ages, the prevalence and annual incidence of OA are steadily rising worldwide. As of 2017, approximately 303 million people globally were reported to suffer from hip and knee OA, making it the 15th-leading cause of disability and a major socioeconomic burden (2, 3). OA is a multifactorial disease, with known risk factors including aging, obesity, metabolic syndrome, female sex, trauma, and genetic predisposition (4). Increasing evidence suggests that OA involves the entire joint, affecting structures such as articular cartilage, subchondral bone, synovium, ligaments, menisci, and the infrapatellar fat pad (IPFP), leading to cartilage loss, subchondral-bone growth and destruction, synovial inflammation, and vascular remodeling (5–7). Currently, no drugs are available that can cure or completely reverse OA. Strategies primarily focus on weight control and the use of NSAIDs to prevent OA progression, with most advanced-OA patients requiring total joint replacement to alleviate symptoms (8, 9). Therefore, a deeper understanding of the pathogenesis of OA and the target molecules involved is crucial. This knowledge will help us identify potential diagnostic or therapeutic biomarkers to achieve early identification and prevention of OA progression (10).

Articular cartilage is an avascular and aneural connective tissue consisting of extracellular matrix (ECM) and chondrocytes (11). It is composed of superficial, middle, deep, and calcified layers. Besides water, ECM contains three major organic components: type II collagen, hyaluronic acid, and proteoglycan matrix. Chondrocytes maintain the homeostasis of these matrix components by regulating the balance between ECM synthesis and degradation (12). When articular cartilage is subjected to adverse microenvironments, such as abnormal mechanical loads or inflammatory mediators (13, 14), chondrocytes exhibit phenotypic changes such as apoptosis, ferroptosis, oxidative stress, cellular senescence, dysregulation of autophagy, inflammation, and accelerated catabolism (11, 15). These changes not only lead to ECM degradation but also induce and exacerbate synovial inflammation through damage-associated molecular patterns (DAMPs) (6), ultimately resulting in the destruction of articular cartilage. Pathological processes in articular cartilage are closely related to the pathogenesis of OA, which suggests that identifying characteristic molecules in OA cartilage might provide opportunities for intervention in the progression of the disease. However, due to the heterogeneity of OA chondrocytes and the functional complexity of different cell subtypes, obtaining reliable and representative cartilage target molecules remains challenging.

With the advancement of bioinformatics and omics technologies, especially single-cell sequencing, high-throughput and highly specific tools have become available for disease research, enabling a comprehensive understanding of cellular heterogeneity. Recent studies on cartilage have resolved different cell subpopulations within healthy and OA cartilage samples at single-cell resolution, revealing their progression trajectories and intercellular-communication patterns in OA (16–19). These chondrocyte subpopulations exhibit distinct biological functions at different stages of OA. In end-stage OA cartilage, fibrocartilage chondrocytes (FC) are regarded as a predominant cell population, positioned at the terminal point of the chondrocyte differentiation trajectory, and are likely to contribute to OA progression (16). This has been supported by several studies. For instance, Li et al. (17) demonstrated a significant increase in the number of FC in hand OA cartilage. In addition, studies by Fan et al. (19) and Sun et al. (18) suggested that FC might represent the terminal stage of OA cartilage differentiation, aligning with the fibrotic phenotype observed in late-stage OA cartilage. The transition from normal cartilage, primarily composed of hyaline cartilage, to late-stage OA cartilage, dominated by FC, may indicate the decline of normal cartilage function during OA progression and its shift towards an abnormally proliferative fibrotic phenotype. A recent study has revealed the potential molecular mechanisms behind the fibrotic transformation of OA cartilage, providing new therapeutic targets for OA treatment strategies. Therefore, we hypothesize that FC may represent a key subpopulation in OA cartilage (20). However, research on OA-related FC and their associated biomarkers remains insufficient.

This study aimed to explore key genes and biomarkers associated with FC in OA by integrating single-cell and microarray omics analyses of cartilage data from public databases. We focused on biological functions of these genes in the pathogenesis of OA. We hope these findings will provide potential targets for identification of and intervention into OA.

## 2 Methods

### 2.1 Download and processing of datasets

We obtained the OA-associated articular-cartilage tissue single-cell dataset GSE255460 from the Gene Expression Omnibus (GEO) database (https://www.ncbi.nlm.nih.gov/geo/). 3 OA cartilage samples and 3 non-OA samples from this dataset were used for further analysis. Simultaneously, we collected 3 independent OA-related articular-cartilage microarray datasets from the GEO database: GSE169077, GSE178557, and GSE117999. The GSE169077 dataset included 6 samples from OA patients and five from non-OA individuals serving as normal controls. The GSE178557 dataset comprised 4 disease samples and 4 normal samples, while the GSE117999 dataset contained 12 disease samples and 12 normal samples. In addition, we acquired the single-cell ribonucleic acid sequencing (scRNA-seq) dataset GSE114007, including 18 control and 20 OA samples, from the GEO database to use as an external-validation dataset for verifying hub genes. Subsequently, we employed the normalizeBetweenArrays and removeBatchEffect functions available in the R package limma (R Foundation for Statistical Computing, Vienna, Austria) to normalize the raw data of each microarray dataset and eliminate batch effects after the merging of three independent datasets.

### 2.2 Single-cell analysis

The Seurat package in R was used to analyze the six articular-cartilage scRNA samples from GSE255460. To ensure cell quality, we set the proportion of red blood cell genes to not exceed 3% and filtered out cells with high mitochondrial-gene proportions (>10%), fewer than 300 or more than 7000 detected genes, and total expression counts greater than 100,000. After normalizing the data, we selected the top 3000 most variable genes using the varianceStabilizingTransformation (vst) function and extracted principal components (PCs) using the RunPCA function. To account for batch effects among samples, we integrated the data using the RunHarmony function. Subsequently, we used the FindNeighbors function for cell clustering and RunUMAP for dimensionality reduction, setting the resolution to 0.8. The FindAllMarkers function was then used to identify marker genes for each cell cluster. Based on a previous study (19), we manually annotated 11 chondrocyte subpopulations. The FeaturePlot function was used to display characteristic markers of the cell clusters and the ggplot2 package to create bar plots. Finally, we identified genes that differentially expressed between groups of FC using the FindMarkers function with the Wilcoxon test and defined these genes as FC-related genes (FCRGs). These results were visualized using a volcano plot.

### 2.3 Differential-expression analysis of genes between OA and normal samples

To analyze differential expression of genes between the OA and control groups, we used the limma package, setting the significance threshold at |log2 fold change| > 0.585 and P < 0.05. This resulted in the identification of 243 differentially expressed genes (DEGs), which we visualized using heatmaps and volcano plots created with the pheatmap and ggplot2 packages, respectively. In addition, the chromosomal locations of the DEGs were visualized using the circlize package.

### 2.4 Functional-enrichment analysis

We performed the Kyoto Encyclopedia of Genes and Genomes (KEGG) and Gene Ontology (GO) functional-enrichment analyses on FCRGs, covering categories such as Biological Process (BP), Molecular Function (MF), and Cellular Component (CC). Subsequently, we retrieved the h.all.v2023.2.Hs.entrez.gmt gene set from the MSigDB website (https://www.gsea-msigdb.org/gsea/msigdb) for GSEA enrichment analysis of DEGs. All analyses were conducted using the clusterProfiler package with the P-value cutoff set to < 0.05. We visualized the results using the enrichplot package. We further performed pathway and process enrichment analysis for DEGs using the Metascape website (21) (https://metascape.org/), selecting KEGG, GO, and Reactome Gene Sets as the datasets. The parameters were set as follows: Min Overlap = 3, P-Value Cutoff = 0.01, and Min Enrichment = 1.5. In subsequent analyses, we utilized the ‘GSVA’ package to conduct GSVA analysis on the top 20 enriched pathways or processes identified from the DEGs. Spearman correlation analysis was performed between the sample scores of each pathway or process and the expression levels of the six biomarkers. The results were visualized using heatmaps and scatter plots.

### 2.5 Weighted gene co-expression network analysis

The Weighted Gene Co-expression Network Analysis (WGCNA) package was used to construct a gene co-expression network for identifying key gene modules associated with OA. First, based on the pre-processed gene expression data, we constructed a sample clustering tree and detected outliers. We then used the PickSoftThreshold function to calculate the optimal soft threshold (β) and converted the similarity matrix into an adjacency matrix. Gene co-expression modules were identified using hierarchical clustering and the dynamic tree cut algorithm, and each was labeled with a different color. Subsequently, we analyzed correlations between gene modules and clinical traits of the samples, identifying the modules most highly associated with OA for further analysis.

### 2.6 Identification and validation of FC-related OA biomarkers using machine learning

Before applying machine learning algorithms, we intersected the FCRGs obtained from scRNA-seq analysis, the DEGs, and the genes from the WGCNA module most closely associated with OA, which resulted in 18 intersecting genes. Intersection results were visualized using Venn diagrams created with the VennDiagram package. Subsequently, we used the glmnet package to apply the least absolute shrinkage and selection operator (LASSO) algorithm, selecting the optimal λ-value based on 10-fold cross-validation. Key genes identified through this process were defined as potential FC-related biomarkers, and their value in OA disease progression and diagnosis was investigated. We validated the expression of these biomarkers in both the training cohort (GSE169077, GSE178557, and GSE117999) and the external-validation cohort (GSE114007). Results were presented as violin and box plots.

### 2.7 Construction and evaluation of an FC-related diagnostic model of OA

We performed multivariable logistic-regression analysis on the key genes identified by the machine learning algorithm to construct an FC-related diagnostic model. The rms package was used to develop the model and create a nomogram for OA. We assessed the diagnostic utility of the key genes and the model using receiver operating characteristic (ROC) curves generated by the pROC package. Calibration curves and decision curve analysis (DCA) were employed to validate the model’s accuracy and clinical utility.

### 2.8 Identification of transcription factor–gene networks

To identify transcription factors (TFs) associated with key genes, we used NetworkAnalyst v3.0 (https://www.networkanalyst.ca/) to predict TFs from the Encyclopedia of Deoxyribonucleic Acid (DNA) Elements (ENCODE) database (22). The results were visualized using Cytoscape software v 3.9.0 (https://cytoscape.org/) to create TF–gene interaction network maps.

### 2.9 Collection of cartilage samples and immunohistochemical staining

We obtained six OA cartilage samples from patients undergoing total knee arthroplasty for knee OA and six normal-cartilage samples from patients undergoing amputation and arthroscopic surgery due to trauma and infection. All patients signed their informed consent before the study. The study was approved by the Ethics Committee of Shantou Central Hospital (Shantou, China), which also supervised the collection and handling of the samples (Ethics Approval No. 2019-047). Details of the clinical samples, including the OA grade, as well as the age and gender of the patients, are presented in **Table 1**.

**Table 1:** Information about the clinical samples.

	Group	Gender	Age	OA grade (Kellgren-Lawrence System)
Patient 1	Normal	Male	18	0
Patient 2	Normal	Male	19	0
Patient 3	Normal	Male	27	0
Patient 4	Normal	Female	37	1
Patient 5	Normal	Female	30	0
Patient 6	Normal	Female	15	0
Patient 7	OA	Male	67	4
Patient 8	OA	Female	59	3
Patient 9	OA	Female	71	4
Patient 10	OA	Female	69	3
Patient 11	OA	Female	69	3
Patient 12	OA	Male	60	3

To validate the expression of three key genes—solute carrier family 7, member 8 (SLC7A8) and adenosine triphosphate (ATP)–binding cassette sub-family A members 5 and 6 (ABCA5, ABCA6)—in our cartilage samples, we performed Safranin O/Fast Green (G1371; Beijing Solarbio Science & Technology Co., Ltd., Beijing, China) and immunohistochemical (IHC) staining. First, tissues fixed in 4% paraformaldehyde were dehydrated, embedded in paraffin, sectioned into 3-μm thick slices, and baked. We placed the deparaffinized slides in ethylenediaminetetraacetic acid (EDTA) retrieval solution and citric acid retrieval solution for antigen retrieval and then incubated them with an endogenous-peroxidase blocker at 37°C for 10 min. Next, 10% goat serum was added to the tissue sections. After 20 min of blocking at 37°C, we applied primary antibodies (Abs): rabbit anti-ABCA5 (1:50; Bioss Antibodies, Woburn, MA, USA), rabbit anti-ABCA6 (1:50; Affinity Biosciences, Cincinnati, OH, USA), and mouse anti–L-type amino acid transporter 2 (LAT2; 1:50; Affinity) at optimal dilutions. (LAT2 is the protein that corresponds with SLC7A8 gene expression.) Then, we incubated the slides in a humid chamber at 4°C overnight (approximately 12–16 h). Appropriate secondary Abs were then added to the tissue sections and incubated at 37°C for 1 h. After color development with 3,3′-diaminobenzidine (DAB) reagent, we counterstained tissues using hematoxylin. Finally, positive cells in the cartilage tissues were counted using ImageJ software (National Institutes of Health, Bethesda, MD, USA). To assess the degree of cartilaginous-tissue damage, we performed Safranin O/Fast Green staining per the kit instructions and evaluated the cartilage tissue according to the OARSI scoring system. For each sample, our histological analysis data were collected from five randomly selected high-power fields. The overall average for each sample was calculated by averaging the values obtained from these selected regions.

### 2.10 Statistical analysis

We used GraphPad Prism v10.0 (GraphPad Software, Inc., San Diego, CA, USA) for statistical analysis and graphing of histological data. Proportions of positive cells in the normal and OA groups were compared using an unpaired t test. P < 0.05 was considered statistically significant.

## 3 Results

### 3.1 Identification and functional-enrichment analysis of fibrocartilage chondrocyte–related genes in OA

**Figure 1:**
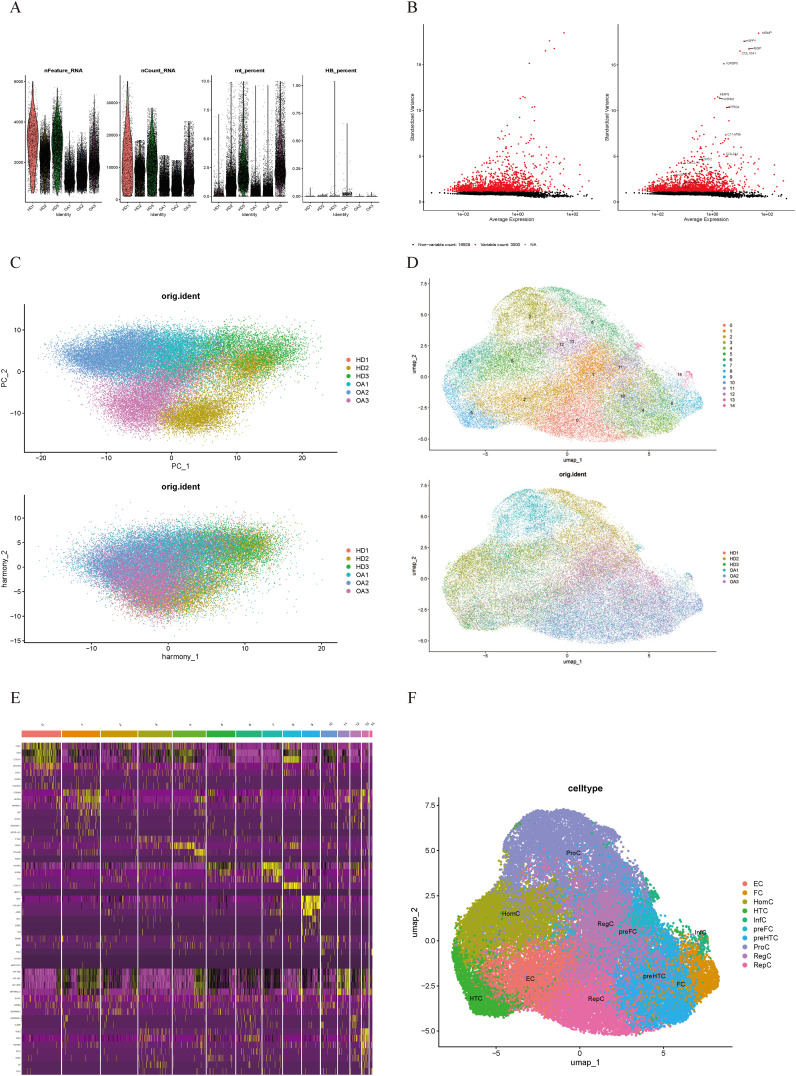
Quality control and cell type annotation of scRNA-seq data from GSE255460. **(A)** Violin plot of filtered scRNA-seq data, showing a total of 45,368 cells meeting the criteria. **(B)** Top 3000 highly variable genes are displayed as red dots in the scatter plot, with non-variable genes represented as black dots. **(C)** Single-cell data after normalization and batch effect correction. **(D)** UMAP plot shows 15 cell clusters. **(E)** Heatmap of marker genes’ expression levels for each cell cluster. **(F)** UMAP plot shows 10 annotated cell clusters.

**Figure 2:**
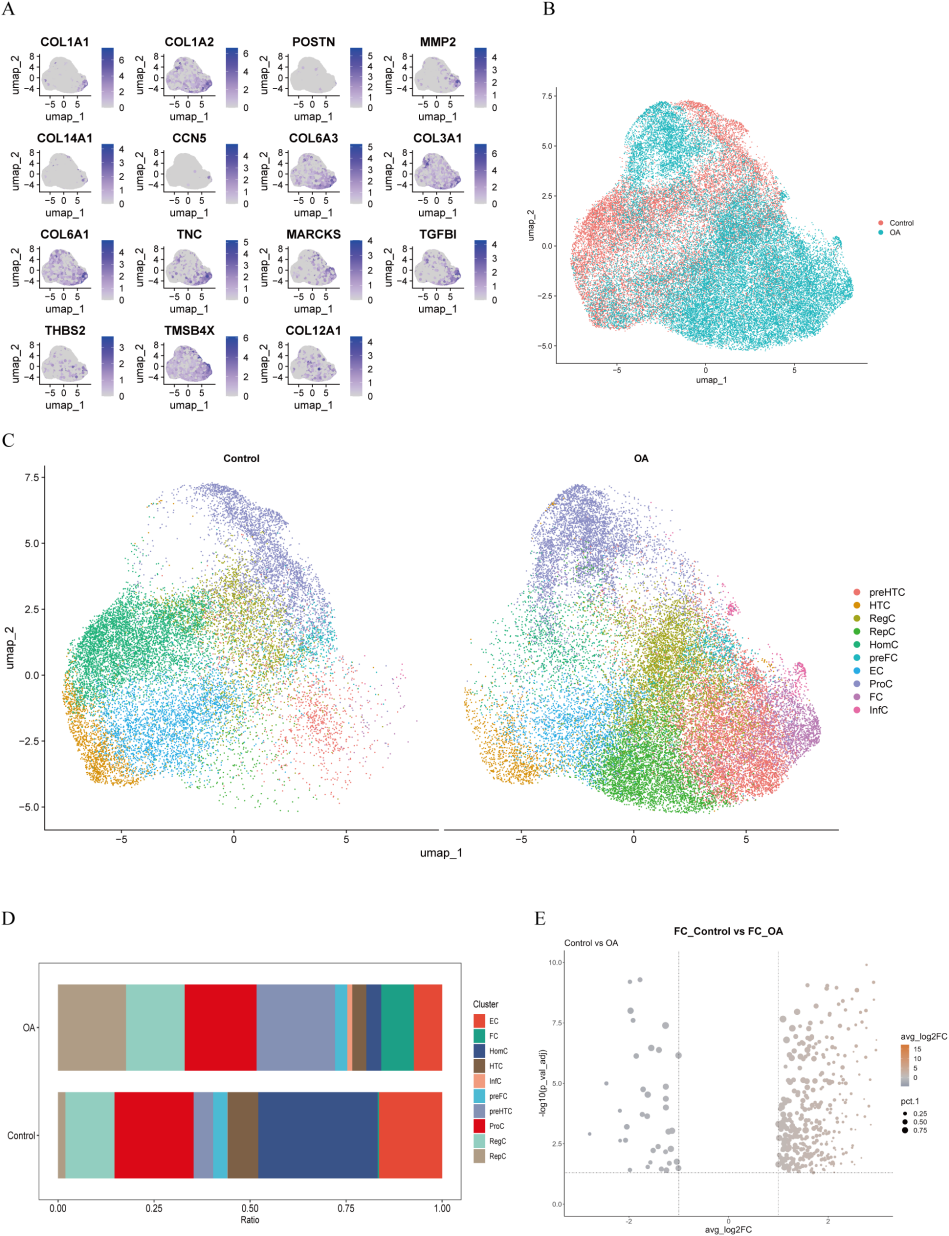
Markers, distribution, proportions, and differentially expressed genes of FC in OA cartilage. **(A)** Distribution of FC-related cell markers at specific locations on UMAP plot. **(B, C)** Distribution of chondrocyte subpopulations in the control and OA groups displayed in the UMAP plot. **(D)** Bar plot showing proportions of cell clusters in the control and OA groups. **(E)** Volcano plot showing FC-related differentially expressed genes (FCRGs) between the control and OA groups. X-axis represents average log2 fold change values; Y-axis represents −log10(Pval_adj) values.

We analyzed the scRNA-seq data from dataset GSE223964. Initially, we performed data quality control, filtering out genes and cells that did not meet the standards in each sample (**Figure 1A**). After normalizing the data, we identified and selected the top 3000 genes exhibiting high variability (**Figure 1B**). Ultimately, we obtained 45,368 cells. We then performed principal-component analysis (PCA) for dimensionality reduction and removed batch effects between samples (**Figure 1C**). We used a resolution of 0.8 for cell clustering and visualized 15 cell subpopulations using the Uniform Manifold Approximation and Projection (UMAP) algorithm (**Figure 1D**), with marker genes of each subpopulation displayed in a heatmap (**Figure 1E**). In accordance with a previous study reporting on cell markers (19), we manually annotated 10 chondrocyte types: homeostatic chondrocytes (HomC), hypertrophic chondrocytes (HTC), reparative chondrocytes (RepC), regulator chondrocytes (RegC), proliferative chondrocytes (ProC), prehypertrophic chondrocytes (preHTC), FC, prefibrocartilage chondrocytes (preFC), inflammatory chondrocytes (InfC), and effector chondrocytes (EC) (**Figure 1F**). The feature markers of FC are showed (**Figure 2A**). Furthermore, we compared cell distribution between OA and normal cartilage and found significant differences (**Figures 2B, C**). These distributions are presented in a bar chart, which shows that the proportion of FC in OA cartilage was significantly higher than that in normal cartilage (**Figure 2D**). Finally, we identified 545 genes that differentially expressed related to FC (FCRGs) (**Figure 2E**), as shown in **Supplementary Data Sheet 2**.

**Figure 3:**
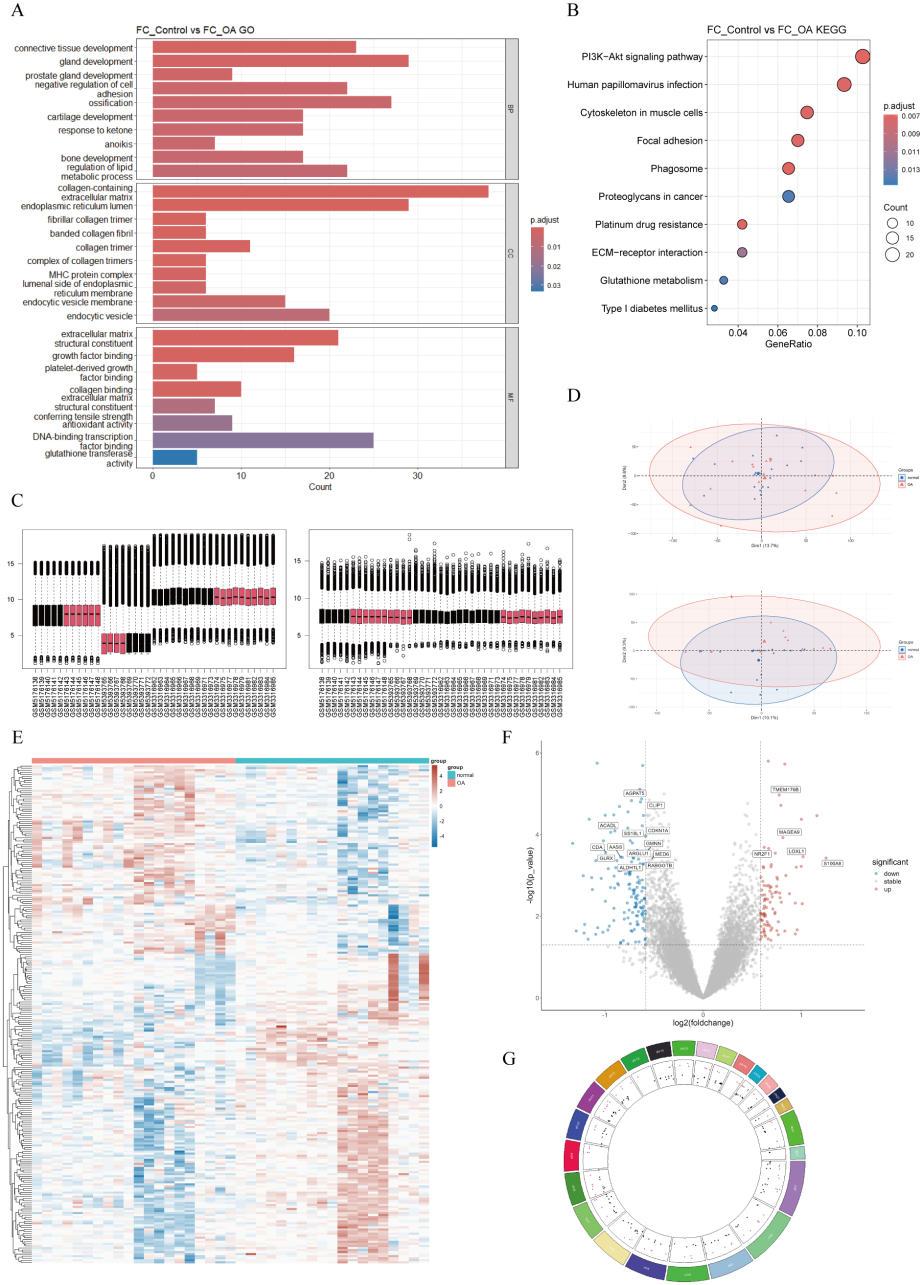
Functional-enrichment analysis of FCRGs and DEGs between normal and OA groups. **(A)** Bar plot of GO enrichment analysis for FCRGs, including BP, CC, and MF categories. X-axis represents the number of genes enriched in each pathway, and color represents Padjust. **(B)** Bubble plot of KEGG enrichment pathways for FCRGs. X-axis represents the ratio of enriched genes, bubble size indicates the number of genes enriched in Biological Processes, and color represents Padjust. **(C, D)** Box plots and PCA, respectively, illustrating integration and batch effect correction of data from GSE169077, GSE178557, and GSE117999. **(E)** Heatmap showing DEGs between normal and OA groups. Colors represent gene expression levels, with upregulated genes shown in red and downregulated genes in blue. **(F)** Volcano plot of DEGs. X-axis represents log2 (fold change); Y-axis represents −log10 (P). Red dots indicate significantly upregulated genes, blue dots indicate significantly downregulated genes, and gray dots represent genes with no significant differential expression. **(G)** Circos plot showing the distribution of DEGs in mitochondria.

To elucidate the functions of these genes, we performed GO annotation and KEGG pathway analysis on them (**Figures 3A, B**). As shown in the bar chart, GO analysis indicated that main terms in the BP category included connective-tissue development, gland development, prostate gland development, negative regulation of cell adhesion, ossification, cartilage development, response to ketone, anoikis, bone development, and regulation of lipid metabolic process. In the CC category, the main terms were collagen-containing ECM, endoplasmic-reticulum lumen, fibrillar-collagen trimer, banded-collagen fibril, collagen trimer, complex of collagen trimers, major histocompatibility complex (MHC) protein complex, luminal side of endoplasmic reticulum membrane, endocytic-vesicle membrane, and endocytic vesicle. MF category terms included ECM structural constituent, growth factor (GF) binding, platelet-derived GF binding, collagen binding, ECM structural constituent conferring tensile strength, antioxidant activity, DNA-binding TF binding, and glutathione transferase activity. As shown in the bubble chart, KEGG pathway analysis revealed that FCRGs were primarily enriched in the phosphatidylinositol-4,5-bisphosphate 3-kinase (PI3K)/protein kinase B (Akt) signaling pathway, human papillomavirus (HPV) infection, focal adhesion, cytoskeleton in muscle cells, phagosome, proteoglycans in cancer, platinum drug resistance, ECM–receptor interaction, glutathione (GSH) metabolism, and type 1 diabetes.

### 3.2 Differential-expression analysis and GSEA analysis between OA and normal groups

**Figure 4:**
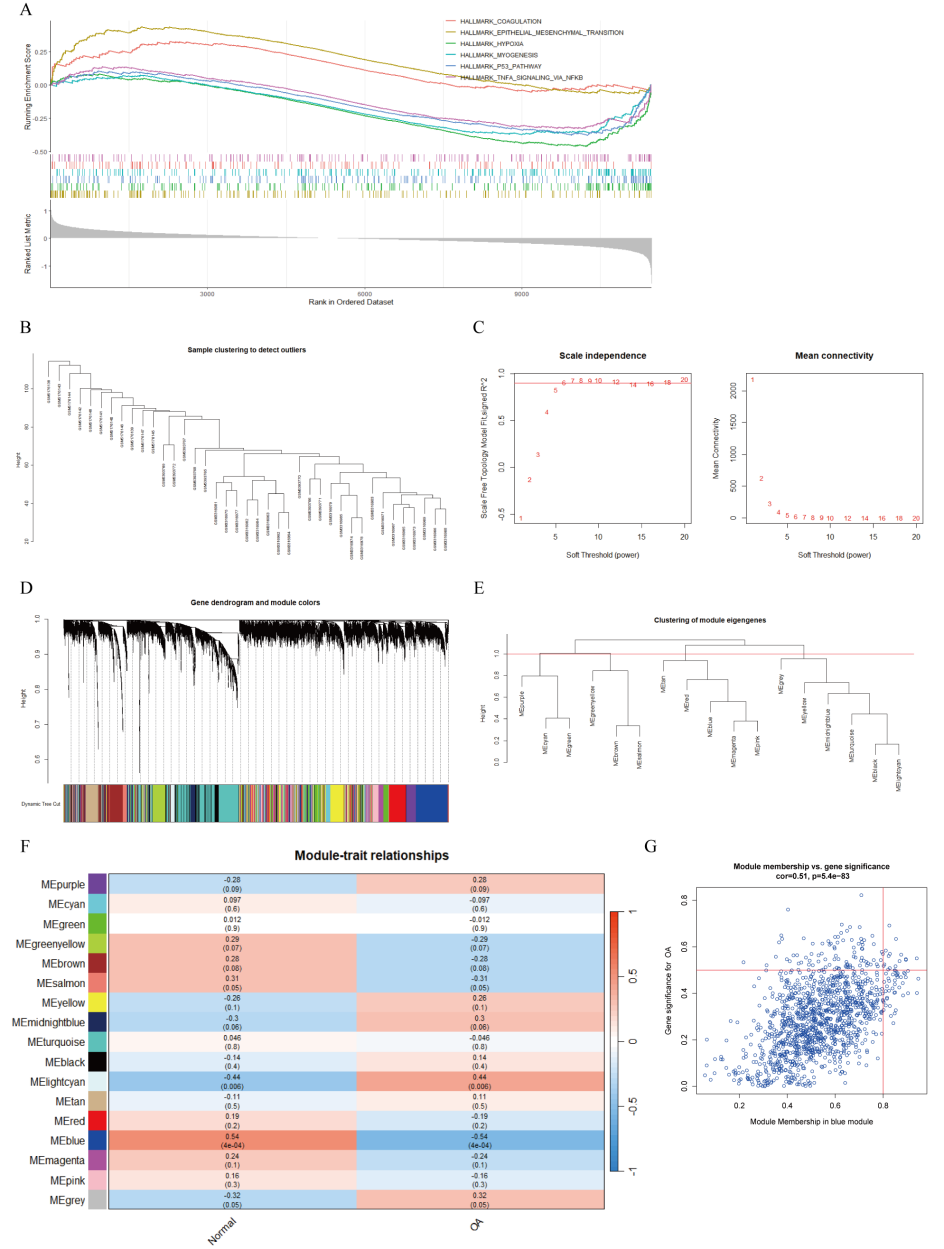
GSEA analysis of DEGs and WGCNA. **(A)** GSEA analysis of DEGs based on h.all.v2023.2.Hs.entrez.gmt gene set. **(B)** Sample clustering dendrogram to remove outlier samples. **(C)** Determination of optimal soft-threshold powers (β) based on scale independence and mean connectivity. **(D)** Gene cluster dendrogram and gene module colors. **(E)** Clustering dendrogram of module eigengenes. **(F)** Heatmap of correlations between module eigengenes and clinical traits, with P-values annotated by both color and numerical value. **(G)** Scatter plot showing correlation between OA and blue module genes.

To identify DEGs between OA and normal cartilage, we integrated and batch-corrected three independent datasets (GSE169077, GSE178557, and GSE117999) to eliminate batch effects between samples (**Figures 3C, D**). A total of 39 samples were obtained, including 20 OA and 19 normal samples. Using the limma package, we then performed differential-expression analysis, which identified 243 DEGs. These DEGs were visualized using volcano plots, heatmaps, and circos plots (**Figures 3E–G**). Subsequently, we conducted GSEA analysis on the DEGs and presented the top six pathways (**Figure 4A**). The results indicated that DEGs were enriched in the following pathways: coagulation, epithelial–mesenchymal transition (EMT), hypoxia, myogenesis, P53, and tumor necrosis factor-α (TNF-α) signaling via nuclear factor κ-light-chain-enhancer of activated B cells (NF-κB). Additionally, we performed pathway enrichment analysis of the DEGs using the Metascape website, exhibiting the top 20 enriched pathways (**Supplementary Figure 1A**).

### 3.3 Weighted gene co-expression network analysis

A total of 39 samples were used to construct the weighted gene co-expression network. Initially, sample clustering indicated that there were no outlier samples, allowing all samples to be included in subsequent analyses (**Figure 4B**). Setting the scale-free topology model fit index threshold to 0.9, we chose β = 6 as the soft threshold, at which the scale-free network performed optimally (**Figure 4C**). Clustering analysis was used to identify highly similar modules, and with dynamic hybrid cutting, we obtained 17 gene modules (**Figures 4D, E**). We then evaluated the associations between OA and the gene modules. The MEblue gene module (n = 1,239) showed the strongest negative correlation with OA (**Figure 4F**). In addition, the scatter plot for this module demonstrated a positive correlation between module membership (MM) and gene significance (GS; cor = 0.51, P = 5.4e−83) (**Figure 4G**). Consequently, we defined the 1,239 genes in the MEblue module as key OA-related genes.

### 3.4 Identification and validation of FC-related OA biomarkers using LASSO regression

**Figure 5:**
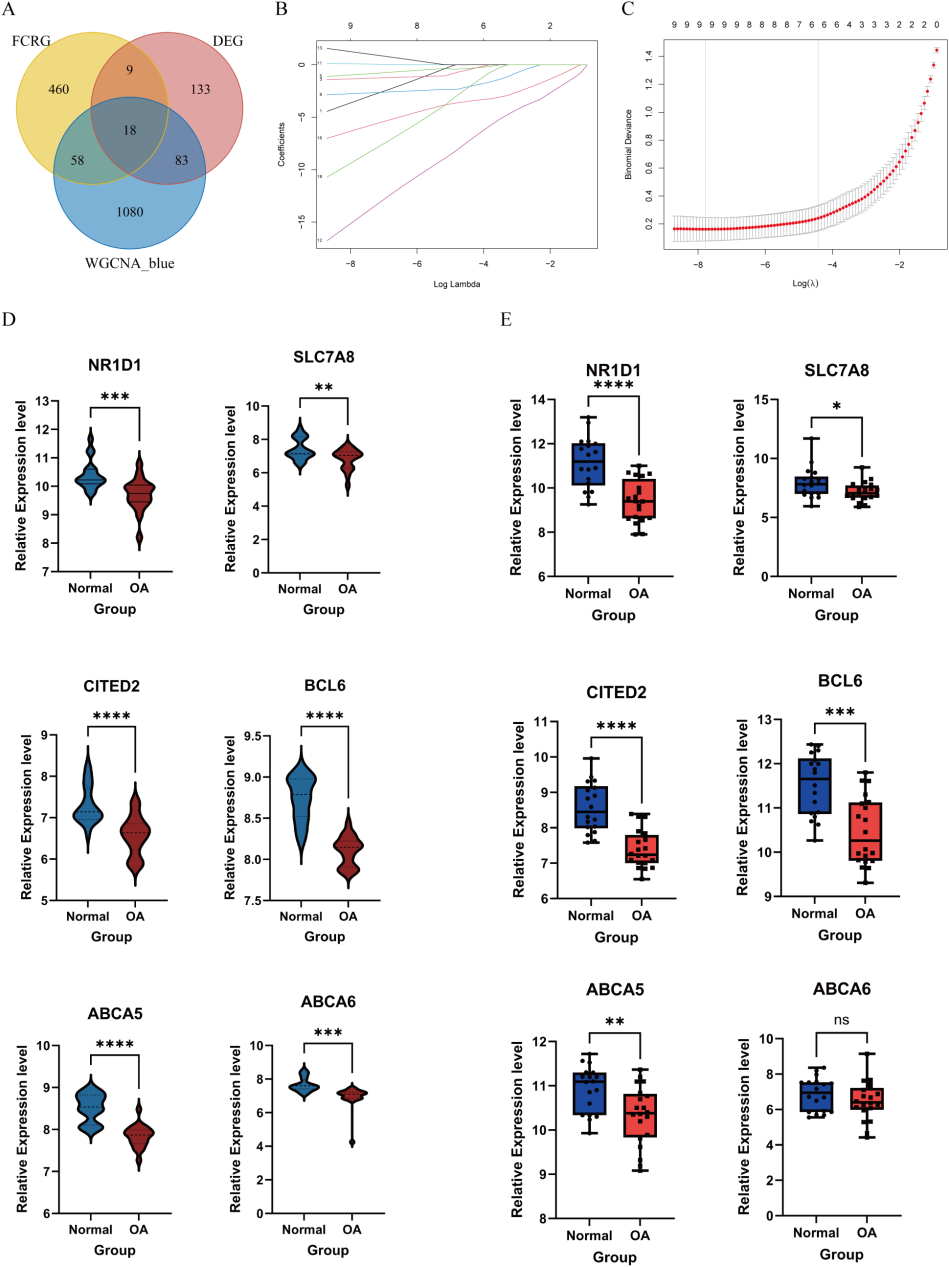
Selection and validation of feature biomarkers of OA. **(A)** Venn diagram showing the intersection of MEblue module genes, FCRGs, and DEGs. **(B, C)** The LASSO regression algorithm was used to select feature biomarkers with minimum log λ. **(D)** Violin plots showing expression levels of six biomarkers in the training datasets (GSE169077, GSE178557, and GSE117999). **(E)** Box plots showing expression levels of six biomarkers in the external-validation dataset (GSE114007). *P < 0.05, **P < 0.01, ***P < 0.001, ****P < 0.0001; ns, not significant.

We intersected the MEblue module genes from the WGCNA analysis with the FCRGs and DEGs (**Figure 5A**). This resulted in the identification of 18 hub genes, on which we performed LASSO regression analysis to identify potential FC-related OA biomarkers. Based on the results of 10-fold cross-validation, we selected six feature genes with minimum log λ (λ = 6) (**Figures 5B, C**). Next, we validated the expression of these six genes in both the training datasets (GSE169077, GSE178557, and GSE117999) and the external-validation dataset (GSE114007). The results showed that in the training datasets, expression levels of the six genes were significantly lower in OA than in normal samples. In the external-validation dataset, expression levels of nuclear receptor subfamily 1 group D member 1 (NR1D1), SLC7A8, Cbp/P300-interacting transactivator with Glu/Asp-rich carboxy-terminal domain 2 (CITED2), B-cell lymphoma 6 (BCL6), and ABCA5 were significantly lower in OA than in normal samples. Meanwhile, ABCA6 showed no statistically significant difference in expression between the two groups (**Figures 5D, E**). Finally, we performed a correlation analysis between the expression levels of the six biomarkers and the characteristics of the DEGs. The analysis revealed that the activities of blood vessel development, extracellular matrix, and assembly of collagen fibrils and other multimeric structures were negatively correlated with the expression of the biomarkers. In contrast, the activities of Forkhead Box (FoxO)-mediated transcription and response to steroid hormone showed positive correlations with the biomarker expression. These results were visualized using both heatmaps and scatter plots (**Supplementary Figures 1B, C**).

### 3.5 Construction and evaluation of an FC-related OA diagnostic model

**Figure 6:**
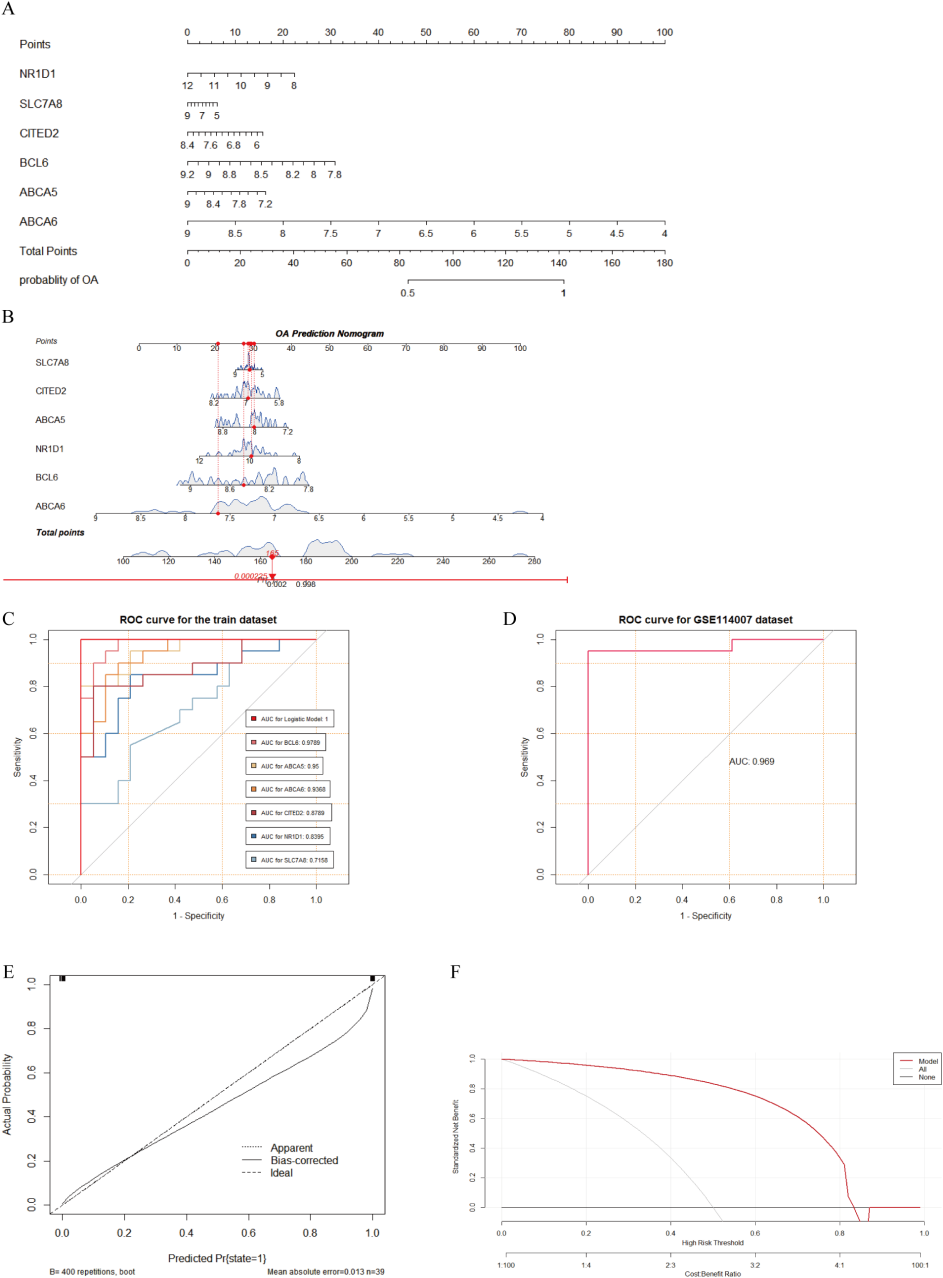
Construction and evaluation of OA diagnostic model based on FC-related biomarkers. **(A, B)** Nomogram constructed for OA based on six FC-related biomarkers. **(C)** ROC curves showing the diagnostic performance of six biomarkers and the model for OA in the training dataset, with AUC values shown in the bottom-right corner. **(D)** ROC curve of the diagnostic model in the GSE114007 dataset, with AUC value shown in the bottom-right corner. **(E)** Calibration curve showing agreement between the model and the ideal model. **(F)** DCA curve indicating potential clinical benefit of the model for decision making.

Based on the six FC-related biomarkers, we constructed an OA diagnostic model using logistic regression and created a nomogram to evaluate the risk of OA (**Figures 6A, B**). ROC curve analysis showed that areas under the curve (AUCs) and 95% confidence intervals (CIs) for BCL6, ABCA5, ABCA6, CITED2, NR1D1, and SLC7A8 were, respectively, 0.9789 (95% CI, 0.9457–1.0000), 0.95 (95% CI, 0.8899–1.0000), 0.9368 (95% CI, 0.8672–1.0000), 0.8789 (95% CI, 0.7668–0.9911), 0.8395 (95% CI, 0.7104–0.9685), and 0.7158 (95% CI, 0.5546–0.8770), indicating that these six biomarkers had significant discriminatory power between OA and normal samples. Furthermore, the diagnostic model achieved an AUC of 0.9694 in the external-validation dataset, confirming its excellent diagnostic capability (**Figures 6C, D**). In addition, the calibration curve demonstrated a high degree of concordance between the model and the ideal model (**Figure 6E**). Based on the DCA results from the external-validation dataset, when the diagnostic threshold for OA is set between 0.1 and 0.7, the net benefit of the model surpasses both the “None” strategy (no intervention for anyone) and the “All” strategy (intervention for everyone). This indicates that the model can effectively distinguish between high-risk and low-risk individuals to some extent, providing better clinical decision-making benefits for OA patients under moderate intervention (**Figure 6F**).

### 3.6 Construction of the TF regulatory networks

**Figure 7:**
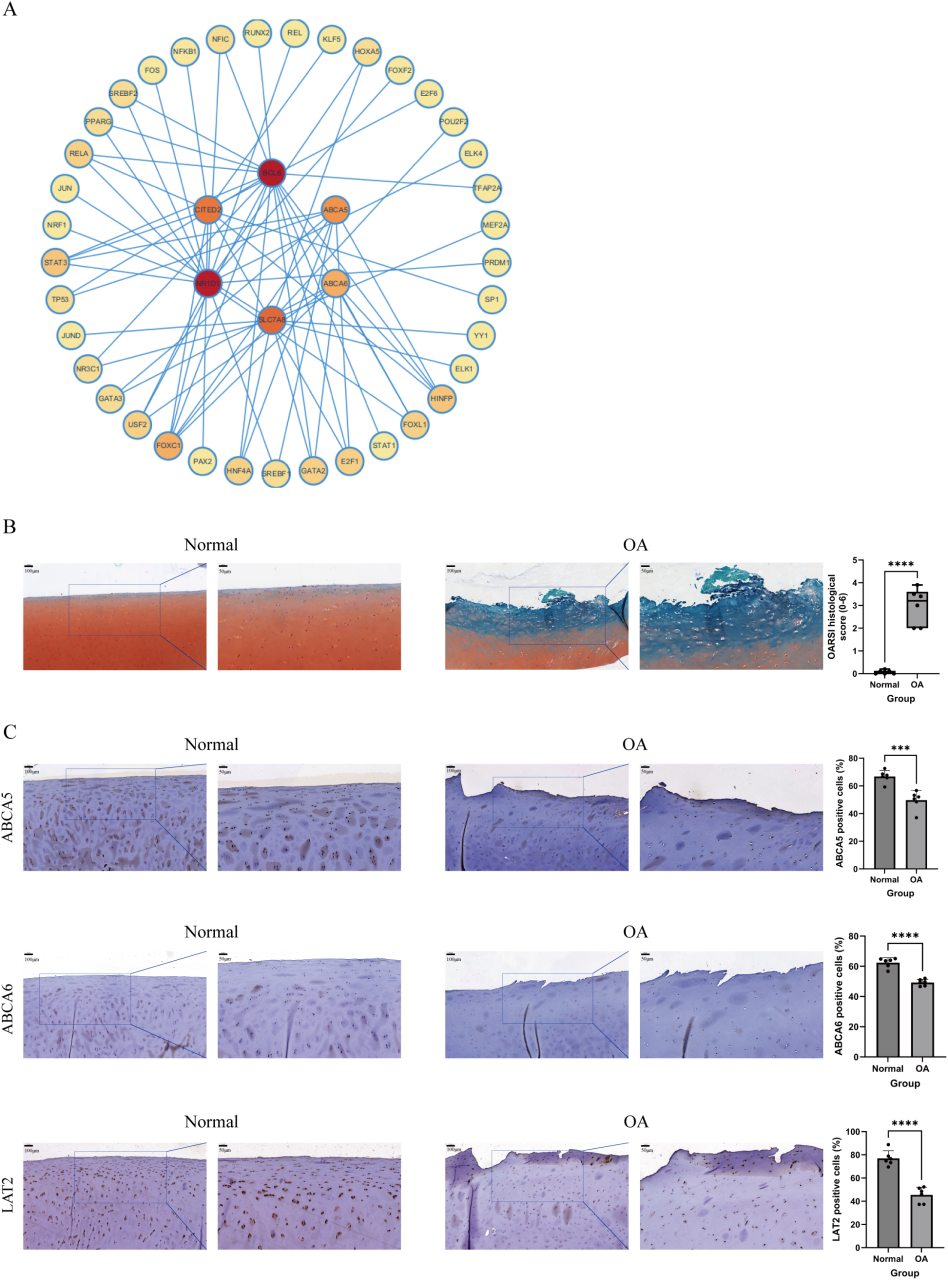
A TF–gene interaction network and validation of feature biomarkers via IHC. **(A)** TF–gene interaction network, with key genes at the center and TFs at the periphery. The darker the color of the TF, the stronger its interaction with the key genes. **(B)** Safranin O/Fast Green staining of cartilage samples from the normal and OA groups and the OARSI score used to evaluate the cartilage lesions in both groups (n = 6 per group). **(C)** IHC staining showing expression levels of ABCA5, ABCA6, and LAT2 in cartilage samples from the normal and OA groups (n = 6 per group). Scale bar: 100 and 50 μm. ***P < 0.001, ****P < 0.0001.

We used the NetworkAnalyst platform and the ENCODE database to predict TFs closely associated with the six key genes (**Figure 7A**). The TF regulatory network was visualized using Cytoscape. In the regulatory network that we built, 37 TFs were identified to interact with the six genes. Notably, forkhead box C1 (FOXC1) was found to interact with all key genes; histone H4 transcription factor (HINFP) interacted with BCL6, CITED2, ABCA5, and ABCA6; and signal transducer and activator of transcription 3 (STAT3) interacted with BCL6, CITED2, NR1D1, and ABCA5.

### 3.7 Validation of OA biomarkers expression in cartilage by IHC staining

We first performed Safranin O/Fast Green staining on cartilage samples from the normal and OA groups and evaluated the degree of cartilage lesions based on the OARSI score (**Figure 7B**). The results showed that the surface of cartilage in the normal group was intact, while cartilage in the OA group exhibited significant loss of cartilage matrix, depletion of Safranin O staining, visible vertical fissures, as well as chondrocyte death and cluster formation. These findings indicated significant degenerative changes in the OA group compared with the normal group. Subsequently, to validate protein expression levels of OA biomarkers in cartilage, we performed IHC staining on samples from both groups. The results demonstrated that expression levels of ABCA5, ABCA6, and LAT2 (the protein product of the SLC7A8 gene) were significantly decreased in the cartilaginous tissue of the OA group compared with that of the normal group, consistent with our analysis of the public datasets (**Figure 7C**).

## 4 Discussion

As understanding of OA’s pathogenesis has deepened, a number of drugs have recently emerged that aim to alter disease progression by modifying OA pathophysiology (23). These drugs, known as disease-modifying osteoarthritis drugs (DMOADs), have shown promising results in cell and animal experiments (24–28). However, none has yet been approved by regulatory agencies for clinical use. This highlights the importance of deepening our understanding of OA pathogenesis, as well as the need for advancements in OA-related imaging and biochemical markers (29, 30). The destruction of articular cartilage, a pathological hallmark of OA, is considered an important event in the progression of the disease, suggesting that OA cartilage might be a reliable target for identifying disease-specific molecules (31, 32). Biological differences among chondrocyte subpopulations have also become a research hotspot in recent years (16, 33, 34). Given these insights, our study focused on cartilage tissue and FC, considering their substantial presence and functional significance in OA cartilage (17, 20, 35). We performed single-cell analysis and microarray analysis on datasets from GEO, employing WGCNA and machine learning algorithms to identify key genes. The pathways closely associated with these genes were subsequently identified. Finally, we exploratively constructed a risk model and conducted experimental validation. These findings can help us elucidate the underlying mechanisms of OA pathogenesis and identify potential therapeutic targets.

Through single-cell analysis of the GSE223964 dataset, we identified 545 marker genes associated with the FC subpopulation. GO analysis revealed that these genes were involved in connective-tissue development, negative regulation of cell adhesion, ossification, anoikis, regulation of lipid metabolic process, collagen-containing ECM, and platelet-derived GF binding. In addition, KEGG analysis showed significant associations with signaling pathways such as PI3K/Akt, focal adhesion, phagosome, ECM–receptor interaction, and GSH metabolism.

Endochondral ossification is typically a critical physiological process in bone growth, but its abnormal activation in OA leads to cartilage degeneration and osteophyte formation, making it a key pathological feature of the disease (36). Chondrocyte hypertrophic differentiation is considered a pivotal step in endochondral ossification, with this phenotypic change representing the loss of normal cartilage function (37). Studies have suggested that hypertrophic chondrocytes in tissue engineering may contribute to the formation of fibrocartilage (38). Our research showed that FCRGs are significantly enriched in the ossification process, which indicates that FC, representing a fibrotic cartilage phenotype, may play a role in cartilage calcification, thereby promoting OA progression. Anoikis, a form of programmed cell death triggered by the loss of proper cell adhesion (39), involves complex signaling pathways and has been implicated in the pathogeneses of various tumors (40). Recent studies have begun exploring the relationship between anoikis and articular diseases. Wang et al. (41) demonstrated that developmentally regulated endothelial-cell locus 1 protein (DEL1) can protect chondrocytes by inhibiting anoikis through integrin signaling. Another study investigated anoikis-related OA genes using single-cell analysis (42), highlighting the connection between anoikis and OA cartilage degeneration. Due to the limited research on anoikis in OA cartilage, its potential negative role in cartilage homeostasis, particularly in relation to FC, may require further exploration. Furthermore, the association between the PI3K/Akt signaling pathway and OA has been extensively studied (43, 44). Activation of this pathway is linked to various BPs in OA cartilage, including ECM metabolism, production of inflammatory mediators, and negative regulation of apoptosis (45). Our analysis suggested that the PI3K/Akt signaling pathway played a significant role in the FC subpopulation in OA. These pathways may provide new insights into the role of FC in OA cartilage.

Our differential analysis of microarray data identified 243 DEGs. Enrichment analysis revealed that DEGs are involved in a wide range of biological processes and signaling pathways, such as response to nutrient levels, assembly of collagen fibrils and other multimeric structures, and FoxO-mediated transcription. These characteristics of DEGs are crucial for understanding the molecular mechanisms and pathogenesis of OA. To further identify genes more closely associated with OA traits, We also conducted WGCNA on the microarray data, identifying the MEblue genes module as the most significantly negatively correlated with OA. Then, we intersected the FCRGs, DEGs, and WGCNA key module genes and performed LASSO regression analysis on the intersecting hub genes, ultimately identifying six feature genes (BCL6, ABCA5, ABCA6, CITED2, NR1D1, and SLC7A8). Differential expression of these genes was validated in both the internal and external datasets, identifying them as potential FC-related OA biomarkers.

How these biomarkers, identified from DEGs, represent the characteristics of DEGs and contribute to the OA disease process remains one of our key focuses. To clarify this complex relationship, we performed a correlation analysis between the six specific genes and the pathways significantly enriched by DEGs. Excitingly, we found a strong association between the selected biomarkers and biological processes related to extracellular matrix, collagen fibril assembly, blood vessel development, response to nutrient levels, and FoxO-mediated transcription. This reflects a high degree of consistency between the biological functions of the biomarkers and the characteristics of the DEGs, providing strong support for the selection of these biomarkers. Specifically, the FoxO family of transcription factors has been widely reported for its crucial role in various biological processes, particularly in cellular senescence, metabolic regulation, autophagy, and stress adaptation, where it is considered a key regulator in maintaining cellular homeostasis (46). Akasaki et al. (47) demonstrated that FoxO expression is significantly reduced in aging articular cartilage, suggesting that the downregulation of FOXO proteins may represent a novel mechanism in OA development. Another study reported that overexpression of FoxO1 reduces cartilage inflammatory mediators and enhances cellular resistance to oxidative stress (48). Studies by Matsuzaki et al. (49) and Lee et al. (50) further emphasized the protective role of FoxO in cartilage and meniscus, respectively, with FoxO downregulation in OA potentially leading to the abnormal expression of genes responsible for cellular homeostasis. Our study confirms a strong positive correlation between FoxO-related pathway activity and the expression levels of the six biomarkers, suggesting a functional role for FoxO in the involvement of these biomarkers in the pathogenesis of OA cartilage. This also implies that these biomarkers may play a critical role in protecting cartilage and slowing the progression of OA.

In OA, BCL6 exhibits certain biological and genetic functions, although the specific pathological processes it participates in remain unclear. A bioinformatics study identified BCL6 as a key hub gene linking knee OA and sarcopenia (51). The data analyses of both Xu et al. (52) and Chen et al. (53) recognize BCL6 as an aging-related OA biomarker with diagnostic potential. CITED2 is another transcriptional regulator known to activate under appropriate mechanical load, thereby playing a protective role in cartilage (54, 55). Other studies have shown it to be important in the IPFP (56) and osteoclastogenesis (57). In our study, expression of CITED2 in OA cartilaginous tissue was significantly downregulated compared with the control group, aligning with the concept that protective factors in cartilage are inhibited in OA. This bioinformatics evidence supported CITED2’s role as a protective mediator in cartilaginous tissue. As a crucial component in the regulation of circadian rhythms, NR1D1 is closely associated with type 2 diabetes (58), tumors (59), metabolic disorders (60), and inflammatory diseases (61). Disruption of circadian rhythms is strongly linked to increased OA susceptibility and might be a potential therapeutic target in OA (62–64). Liu et al. (61) revealed that NR1D1 plays a key regulatory role in synovial inflammation and bone destruction in RA. Akagi et al. (65) confirmed downregulation of NR1D1 in OA cartilage, which might impact transforming growth factor-β (TGF-β) signaling in chondrocytes; this is consistent with our study’s findings. SLC7A8, also known as LAT2, is a Na+-independent amino acid transporter widely expressed in human tissues such as those of the small intestine, kidney, placenta, and skeletal muscle, mediating the specific exchange of neutral L-amino acids (66, 67). SLC7A8 might activate mammalian target of rapamycin (mTOR) through glutamine metabolism regulation, contributing to metabolic reprogramming (68). Another member of the SLC7 family, SLC7A5, has been reported to regulate expression levels of matrix metalloproteinases 3 and 13 (MMP-3, MMP-13) in RA synovial cells (69). Another bioinformatics study identified SLC7A5 as an OA biomarker (70).

ABCA5 and ABCA6, members of the ATP-binding cassette transporter superfamily, are located on human chromosome 17q24 and are primarily responsible for lipid transport, particularly cholesterol transport (71–73). Knockout of murine ABCA5 leads to lysosomal-disease–like symptoms (74). DeStefano et al. (75) and Palmer et al. (76) identified ABCA5 as a key gene in hair growth, with ABCA5 knockdown leading to cholesterol homeostasis disruption. Fu et al. (77) confirmed a close association between ABCA5 and late-onset Alzheimer disease, potentially due to its role in maintaining brain cholesterol homeostasis. Another study identified ABCA5 as a mediator of cholesterol efflux in macrophages under high-cholesterol conditions, highlighting its potential in lipid management and atherosclerosis. ABCA6 has been reported to be involved in the progression of various malignancies through cholesterol transport regulation (78). A large-scale genomic study found a 3.65-fold increase in ABCA6 mutation frequency in the Dutch population (79). Gai et al. (80) demonstrated that ABCA6 is a novel target gene of FoxO in human endothelial cells (ECs) and responds to cholesterol levels. This finding strongly supports the close association between the FoxO pathway and OA-related biomarkers revealed in our previous results.

Given the significantly decreased expression of these biomarkers in OA cartilage, we speculate that these key genes may be closely involved in maintaining chondrocyte homeostasis, participating in crucial physiological functions such as cholesterol transport, glutamine metabolism, and circadian rhythm regulation. The reduced expression of these biomarkers may disrupt chondrocyte homeostasis, thereby promoting OA progression. Specifically, we hypothesize that the low expression of ABCA5 and ABCA6 in OA cartilage may be regulated by FoxO and potentially disrupt intracellular cholesterol homeostasis by inhibiting cholesterol transport. This imbalance in intracellular cholesterol could ultimately contribute to the pathological progression of OA cartilage. Recently, the association between OA and impaired lipid metabolism has garnered increasing attention (81, 82). Choi et al. (83) experimentally confirmed that cholesterol regulates chondrocytes in OA through the cholesterol 25-hydroxylase (CH25H)/cytochrome P450 7B1 (CYP7B1)/retinoic acid receptor (RAR)–related orphan receptor-α (RORα) axis. Another study has revealed a correlation between cholesterol and cartilage degeneration (84). In addition, ABCA1, a member of the ABCA subfamily and a cholesterol efflux gene, has been shown to be downregulated in OA cartilage (85). These insights may help uncover the complex pathological mechanisms involved in OA cartilage degeneration and provide potential therapeutic targets for OA. However, more comprehensive data analysis and further experimental models are needed to validate this hypothesis and explore the underlying complex molecular mechanisms.

Based on these biomarkers, we have exploratively constructed a new OA risk model based on cartilage RNA-seq data. The diagnostic ability and reliability of the model and of each biomarker were subsequently evaluated. AUC values for each biomarker were > 0.7, with that of BCL6 as high as 0.9789, demonstrating their ability to distinguish between OA and Normal group samples. The DCA result indicated that the model may provide potential support for clinical decision-making in the identification of OA patients. However, the model is still far from clinical application, and its reliability needs to be validated using larger cartilage sequencing datasets. Compared to other biomarkers such as X-rays and synovial fluid, the acquisition of cartilage samples and subsequent RNA sequencing may involve higher costs and more invasive clinical interventions. Furthermore, due to the uneven distribution of cartilage degeneration in OA, these cartilage-derived biomarkers may be influenced by the sampling region, potentially introducing bias into the predictive outcomes. These limitations may restrict the practical application of the model and suggest that constructing a predictive model based on a combination of multiple biomarkers could be a direction for future research. However, these exploratory results still hold potential value in expanding the knowledge of diagnostic biomarkers for OA.

To identify molecules involved in the regulation of key genes, we constructed a TF–gene regulatory network. We found that FOXC1, HINFP, and STAT3 were closely related to the key genes, suggesting that complex interactions might be involved in the pathogenesis of OA. However, further research is needed to verify this hypothesis. Finally, we validated protein expression levels of the three key genes—ABCA5, ABCA6, and SLC7A8—in cartilage samples collected from clinical cases. As expected, protein expression levels of these three key genes were significantly lower in the OA than in the normal group, further confirming the reliability of the OA biomarkers we identified.

As previously noted, FC is the primary cell population in OA cartilage and has a strong association with cartilage fibrosis and degeneration. Our analysis further corroborates these findings. In our study, the six FC-associated OA biomarkers identified all showed lower expression levels in OA cartilage, which contrasts with the increased abundance of FC in OA. We hypothesize that during OA progression, the downregulation or even inactivation of genes involved in maintaining normal chondrocyte metabolism and circadian rhythm may disrupt chondrocyte homeostasis, leading to phenotypic changes, increased FC abundance, and ultimately cartilage degeneration. One study demonstrated that the gene expression profile of FC underwent extensive alterations in hand OA and that the proportion of FC associated with increased cartilage degradation was significantly elevated in OA (17). These studies support our hypothesis and help to reveal the specific molecular mechanisms linking biomarkers and FC, although further data and experimental validation are still needed.

Despite its potential insights into disease-related molecules, this study had several limitations. First, the microarray datasets included in our research were sourced from the GEO database, with only a limited number of cartilage samples meeting study requirements. This might have introduced bias into the results. Second, since the dataset does not include data related to X-rays or other biomarkers, our OA model may lack the ability to compare with other biomarkers. In future studies, we aim to incorporate more OA-related biomarkers to enhance the diagnostic efficacy of the model. Moreover, the study validated expression levels of biomarkers through IHC only. The heterogeneity in cartilage degeneration may cause sampling variations that affect the expression levels of these biomarkers in different samples. It is necessary to collect more clinical cases and further explore the potential mechanisms of these biomarkers through *in vivo* and *in vitro* experiments. Additionally, the TF-gene interaction network we constructed was based on connectivity between entities, which may not accurately reflect the precise molecular regulatory relationships. Moreover, the referenced database may not fully capture specific biological phenomena. Therefore, the regulatory relationships within these interaction networks require further validation through experimental data or supporting literature evidence.

## 5 Conclusion

In conclusion, this study employed a multi-omics integrative bioinformatics approach, focusing on the FC subpopulation in OA cartilage, and identified six genes (BCL6, ABCA5, ABCA6, CITED2, NR1D1, and SLC7A8) as FC-related OA biomarkers. An exploratory OA risk model based on cartilage RNA-seq data was also developed. Furthermore, functional enrichment analysis confirmed a strong association between the FoxO pathway and these biomarkers. We hypothesize that ABCA5 and ABCA6 may play a key role in maintaining cholesterol homeostasis in chondrocytes, and their downregulation in OA cartilage could represent a potential molecular mechanism driving cartilage degeneration and fibrosis. These findings are the first to report a link between ABCA family members and OA, potentially advancing our understanding of OA pathogenesis and providing new therapeutic targets for the disease.

## Data Availability

The original contributions presented in the study are included in the article/**Supplementary Material**. Further inquiries can be directed to the corresponding author/s.

## References

[1] Martel-Pelletier J Barr AJ Cicuttini FM Conaghan PG Cooper C Goldring MB. Osteoarthritis. Nat Rev Dis Primers. (2016) 2:16072. doi: 10.1038/nrdp.2016.72 27734845

[2] Safiri S Kolahi AA Smith E Hill C Bettampadi D Mansournia MA. Global, regional and national burden of osteoarthritis 1990-2017: a systematic analysis of the Global Burden of Disease Study 2017. Ann Rheum Dis. (2020) 79:819–28. doi: 10.1136/annrheumdis-2019-216515 32398285

[3] Leifer VP Katz JN Losina E. The burden of OA-health services and economics. Osteoarthritis Cartilage. (2022) 30:10–6. doi: 10.1016/j.joca.2021.05.007 PMC8605034 34023527

[4] Wang T He C. Pro-inflammatory cytokines: The link between obesity and osteoarthritis. Cytokine Growth Factor Rev. (2018) 44:38–50. doi: 10.1016/j.cytogfr.2018.10.002 30340925

[5] Mobasheri A Rayman MP Gualillo O Sellam J van der Kraan P Fearon U. The role of metabolism in the pathogenesis of osteoarthritis. Nat Rev Rheumatol. (2017) 13:302–11. doi: 10.1038/nrrheum.2017.50 28381830

[6] Zhang H Cai D Bai X. Macrophages regulate the progression of osteoarthritis. Osteoarthritis Cartilage. (2020) 28:555–61. doi: 10.1016/j.joca.2020.01.007 31982565

[7] Yao Q Wu X Tao C Gong W Chen M Qu M. Osteoarthritis: pathogenic signaling pathways and therapeutic targets. Signal Transduct Target Ther. (2023) 8:56. doi: 10.1038/s41392-023-01330-w 36737426 PMC9898571

[8] Kolasinski SL Neogi T Hochberg MC Oatis C Guyatt G Block J. 2019 American college of rheumatology/Arthritis foundation guideline for the management of osteoarthritis of the hand, hip, and knee. Arthritis Rheumatol. (2020) 72:220–33. doi: 10.1002/art.41142 PMC10518852 31908163

[9] Katz JN Arant KR Loeser RF. Diagnosis and treatment of hip and knee osteoarthritis: A review. Jama. (2021) 325:568–78. doi: 10.1001/jama.2020.22171 PMC8225295 33560326

[10] Kraus VB Hsueh MF. Molecular biomarker approaches to prevention of post-traumatic osteoarthritis. Nat Rev Rheumatol. (2024) 20:272–89. doi: 10.1038/s41584-024-01102-y 38605249

[11] Goldring SR Goldring MB. Changes in the osteochondral unit during osteoarthritis: structure, function and cartilage-bone crosstalk. Nat Rev Rheumatol. (2016) 12:632–44. doi: 10.1038/nrrheum.2016.148 27652499

[12] Aicher WK Rolauffs B. The spatial organisation of joint surface chondrocytes: review of its potential roles in tissue functioning, disease and early, preclinical diagnosis of osteoarthritis. Ann Rheum Dis. (2014) 73:645–53. doi: 10.1136/annrheumdis-2013-204308 24363359

[13] Jiang W Liu H Wan R Wu Y Shi Z Huang W. Mechanisms linking mitochondrial mechanotransduction and chondrocyte biology in the pathogenesis of osteoarthritis. Ageing Res Rev. (2021) 67:101315. doi: 10.1016/j.arr.2021.101315 33684550

[14] Hodgkinson T Kelly DC Curtin CM O’Brien FJ. Mechanosignalling in cartilage: an emerging target for the treatment of osteoarthritis. Nat Rev Rheumatol. (2022) 18:67–84. doi: 10.1038/s41584-021-00724-w 34934171

[15] Sun K Guo J Guo Z Hou L Liu H Hou Y. The roles of the Hippo-YAP signalling pathway in Cartilage and Osteoarthritis. Ageing Res Rev. (2023) 90:102015. doi: 10.1016/j.arr.2023.102015 37454824

[16] Ji Q Zheng Y Zhang G Hu Y Fan X Hou Y. Single-cell RNA-seq analysis reveals the progression of human osteoarthritis. Ann Rheum Dis. (2019) 78:100–10. doi: 10.1136/annrheumdis-2017-212863 PMC6317448 30026257

[17] Li H Jiang X Xiao Y Zhang Y Zhang W Doherty M. Combining single-cell RNA sequencing and population-based studies reveals hand osteoarthritis-associated chondrocyte subpopulations and pathways. Bone Res. (2023) 11:58. doi: 10.1038/s41413-023-00292-7 37914703 PMC10620170

[18] Sun Z Yan M Wang J Zhang H Ji X Xiao Y. Single-cell RNA sequencing reveals different chondrocyte states in femoral cartilage between osteoarthritis and healthy individuals. Front Immunol. (2024) 15:1407679. doi: 10.3389/fimmu.2024.1407679 38868774 PMC11167083

[19] Fan Y Bian X Meng X Li L Fu L Zhang Y. Unveiling inflammatory and prehypertrophic cell populations as key contributors to knee cartilage degeneration in osteoarthritis using multi-omics data integration. Ann Rheum Dis. (2024) 83:926–44. doi: 10.1136/ard-2023-224420 PMC11187367 38325908

[20] Liu Q Han M Wu Z Fu W Ji J Liang Q. DDX5 inhibits hyaline cartilage fibrosis and degradation in osteoarthritis via alternative splicing and G-quadruplex unwinding. Nat Aging. (2024) 4:664–80. doi: 10.1038/s43587-024-00624-0 PMC11108786 38760576

[21] Zhou Y Zhou B Pache L Chang M Khodabakhshi AH Tanaseichuk O. Metascape provides a biologist-oriented resource for the analysis of systems-level datasets. Nat Commun. (2019) 10:1523. doi: 10.1038/s41467-019-09234-6 30944313 PMC6447622

[22] Zhou G Soufan O Ewald J Hancock REW Basu N Xia J. NetworkAnalyst 3.0: a visual analytics platform for comprehensive gene expression profiling and meta-analysis. Nucleic Acids Res. (2019) 47:W234–w41. doi: 10.1093/nar/gkz240 PMC6602507 30931480

[23] Cho Y Jeong S Kim H Kang D Lee J Kang SB. Disease-modifying therapeutic strategies in osteoarthritis: current status and future directions. Exp Mol Med. (2021) 53:1689–96. doi: 10.1038/s12276-021-00710-y PMC8640059 34848838

[24] Chen P Liu X Gu C Zhong P Song N Li M. A plant-derived natural photosynthetic system for improving cell anabolism. Nature. (2022) 612:546–54. doi: 10.1038/s41586-022-05499-y PMC9750875 36477541

[25] Yan J Shen M Sui B Lu W Han X Wan Q. Autophagic LC3(+) calcified extracellular vesicles initiate cartilage calcification in osteoarthritis. Sci Adv. (2022) 8:eabn1556. doi: 10.1126/sciadv.abn1556 35544558 PMC9094669

[26] Wang X Cai Y Wu C Liang J Tang K Lin Z. Conversion of senescent cartilage into a pro-chondrogenic microenvironment with antibody-functionalized copper sulfate nanoparticles for efficient osteoarthritis therapy. J Nanobiotechnology. (2023) 21:258. doi: 10.1186/s12951-023-02036-5 37550685 PMC10408088

[27] DeJulius CR Walton BL Colazo JM d’Arcy R Francini N Brunger JM. Engineering approaches for RNA-based and cell-based osteoarthritis therapies. Nat Rev Rheumatol. (2024) 20:81–100. doi: 10.1038/s41584-023-01067-4 38253889 PMC11129836

[28] Ma K Pham T Wang J OS I DiCamillo A Du S. Nanoparticle-based inhibition of vascular endothelial growth factor receptors alleviates osteoarthritis pain and cartilage damage. Sci Adv. (2024) 10:eadi5501. doi: 10.1126/sciadv.adi5501 38354243 PMC10866538

[29] Glyn-Jones S Palmer AJ Agricola R Price AJ Vincent TL Weinans H. Osteoarthritis. Lancet. (2015) 386:376–87. doi: 10.1016/s0140-6736(14)60802-3 25748615

[30] Nielsen RL Monfeuga T Kitchen RR Egerod L Leal LG Schreyer ATH. Data-driven identification of predictive risk biomarkers for subgroups of osteoarthritis using interpretable machine learning. Nat Commun. (2024) 15:2817. doi: 10.1038/s41467-024-46663-4 38561399 PMC10985086

[31] Tuerlings M Janssen GMC Boone I van Hoolwerff M Rodriguez Ruiz A Houtman E. WWP2 confers risk to osteoarthritis by affecting cartilage matrix deposition via hypoxia associated genes. Osteoarthritis Cartilage. (2023) 31:39–48. doi: 10.1016/j.joca.2022.09.009 36208715

[32] Peng R Shang J Jiang N Chi-Jen H Gu Y Xing B. Klf10 is involved in extracellular matrix calcification of chondrocytes alleviating chondrocyte senescence. J Transl Med. (2024) 22:52. doi: 10.1186/s12967-023-04666-7 38217021 PMC10790269

[33] Di J Chen Z Wang Z He T Wu D Weng C. Cartilage tissue from sites of weight bearing in patients with osteoarthritis exhibits a differential phenotype with distinct chondrocytes subests. RMD Open. (2023) 9:e003255. doi: 10.1136/rmdopen-2023-003255 37848267 PMC10582868

[34] Kang X Zhang K Wang Y Zhao Y Lu Y. Single-cell RNA sequencing analysis of human chondrocytes reveals cell-cell communication alterations mediated by interactive signaling pathways in osteoarthritis. Front Cell Dev Biol. (2023) 11:1099287. doi: 10.3389/fcell.2023.1099287 37082621 PMC10112522

[35] Housmans BAC Neefjes M Surtel DAM Vitík M Cremers A van Rhijn LW. Synovial fluid from end-stage osteoarthritis induces proliferation and fibrosis of articular chondrocytes via MAPK and RhoGTPase signaling. Osteoarthritis Cartilage. (2022) 30:862–74. doi: 10.1016/j.joca.2021.12.015 35176481

[36] van der Kraan PM Blaney Davidson EN Blom A van den Berg WB. TGF-beta signaling in chondrocyte terminal differentiation and osteoarthritis: modulation and integration of signaling pathways through receptor-Smads. Osteoarthritis Cartilage. (2009) 17:1539–45. doi: 10.1016/j.joca.2009.06.008 19583961

[37] Rim YA Nam Y Ju JH. The role of chondrocyte hypertrophy and senescence in osteoarthritis initiation and progression. Int J Mol Sci. (2020) 21:2358. doi: 10.3390/ijms21072358 32235300 PMC7177949

[38] Cao Z Bai Y Liu C Dou C Li J Xiang J. Hypertrophic differentiation of mesenchymal stem cells is suppressed by xanthotoxin via the p38−MAPK/HDAC4 pathway. Mol Med Rep. (2017) 16:2740–6. doi: 10.3892/mmr.2017.6886 PMC5548016 28677757

[39] Gilmore AP. Anoikis. Cell Death Differ. (2005) 12 Suppl 2:1473–7. doi: 10.1038/sj.cdd.4401723 16247493

[40] Rennebeck G Martelli M Kyprianou N. Anoikis and survival connections in the tumor microenvironment: is there a role in prostate cancer metastasis? Cancer Res. (2005) 65:11230–5. doi: 10.1158/0008-5472.Can-05-2763 PMC2367317 16357123

[41] Wang Z Boyko T Tran MC LaRussa M Bhatia N Rashidi V. DEL1 protects against chondrocyte apoptosis through integrin binding. J Surg Res. (2018) 231:1–9. doi: 10.1016/j.jss.2018.04.066 30278915

[42] Zhang JS Pan RS Li GL Teng JX Zhao HB Zhou CH. Comprehensive analysis of anoikis-related genes in diagnosis osteoarthritis: based on machine learning and single-cell RNA sequencing data. Artif Cells Nanomed Biotechnol. (2024) 52:156–74. doi: 10.1080/21691401.2024.2318210 38423139

[43] Cheng Q Chen M Liu M Chen X Zhu L Xu J. Semaphorin 5A suppresses ferroptosis through activation of PI3K-AKT-mTOR signaling in rheumatoid arthritis. Cell Death Dis. (2022) 13:608. doi: 10.1038/s41419-022-05065-4 35835748 PMC9283415

[44] Xu K He Y Moqbel SAA Zhou X Wu L Bao J. SIRT3 ameliorates osteoarthritis via regulating chondrocyte autophagy and apoptosis through the PI3K/Akt/mTOR pathway. Int J Biol Macromol. (2021) 175:351–60. doi: 10.1016/j.ijbiomac.2021.02.029 33556400

[45] Sun K Luo J Guo J Yao X Jing X Guo F. The PI3K/AKT/mTOR signaling pathway in osteoarthritis: a narrative review. Osteoarthritis Cartilage. (2020) 28:400–9. doi: 10.1016/j.joca.2020.02.027 32081707

[46] Rodriguez-Colman MJ Dansen TB Burgering BMT. FOXO transcription factors as mediators of stress adaptation. Nat Rev Mol Cell Biol. (2024) 25:46–64. doi: 10.1038/s41580-023-00649-0 37710009

[47] Akasaki Y Hasegawa A Saito M Asahara H Iwamoto Y Lotz MK. Dysregulated FOXO transcription factors in articular cartilage in aging and osteoarthritis. Osteoarthritis Cartilage. (2014) 22:162–70. doi: 10.1016/j.joca.2013.11.004 PMC3932989 24269635

[48] Ohzono H Hu Y Nagira K Kanaya H Okubo N Olmer M. Targeting FoxO transcription factors with HDAC inhibitors for the treatment of osteoarthritis. Ann Rheum Dis. (2023) 82:262–71. doi: 10.1136/ard-2021-221269 PMC11005918 36109140

[49] Matsuzaki T Alvarez-Garcia O Mokuda S Nagira K Olmer M Gamini R. FoxO transcription factors modulate autophagy and proteoglycan 4 in cartilage homeostasis and osteoarthritis. Sci Transl Med. (2018) 10:eaan0746. doi: 10.1126/scitranslmed.aan0746 29444976 PMC6204214

[50] Lee KI Choi S Matsuzaki T Alvarez-Garcia O Olmer M Grogan SP. FOXO1 and FOXO3 transcription factors have unique functions in meniscus development and homeostasis during aging and osteoarthritis. Proc Natl Acad Sci U.S.A. (2020) 117:3135–43. doi: 10.1073/pnas.1918673117 PMC7022148 31980519

[51] Yang J Jiang T Xu G Wang S Liu W. Exploring molecular mechanisms underlying the pathophysiological association between knee osteoarthritis and sarcopenia. Osteoporos Sarcopenia. (2023) 9:99–111. doi: 10.1016/j.afos.2023.08.005 37941536 PMC10627980

[52] Xu L Wang Z Wang G. Screening of biomarkers associated with osteoarthritis aging genes and immune correlation studies. Int J Gen Med. (2024) 17:205–24. doi: 10.2147/ijgm.S447035 PMC10807283 38268862

[53] Chen Z Wang W Hua Y. Identification and validation of BCL6 and VEGFA as biomarkers and ageing patterns correlating with immune infiltrates in OA progression. Sci Rep. (2023) 13:2558. doi: 10.1038/s41598-023-28000-9 36781858 PMC9925801

[54] He Z Leong DJ Zhuo Z Majeska RJ Cardoso L Spray DC. Strain-induced mechanotransduction through primary cilia, extracellular ATP, purinergic calcium signaling, and ERK1/2 transactivates CITED2 and downregulates MMP-1 and MMP-13 gene expression in chondrocytes. Osteoarthritis Cartilage. (2016) 24:892–901. doi: 10.1016/j.joca.2015.11.015 26687824

[55] He Z Leong DJ Xu L Hardin JA Majeska RJ Schaffler MB. CITED2 mediates the cross-talk between mechanical loading and IL-4 to promote chondroprotection. Ann N Y Acad Sci. (2019) 1442:128–37. doi: 10.1111/nyas.14021 PMC6956611 30891766

[56] Liu L He Z Xu L Lu L Feng H Leong DJ. CITED2 mediates the mechanical loading-induced suppression of adipokines in the infrapatellar fat pad. Ann N Y Acad Sci. (2019) 1442:153–64. doi: 10.1111/nyas.14025 30891782

[57] Tsukasaki M Huynh NC Okamoto K Muro R Terashima A Kurikawa Y. Stepwise cell fate decision pathways during osteoclastogenesis at single-cell resolution. Nat Metab. (2020) 2:1382–90. doi: 10.1038/s42255-020-00318-y 33288951

[58] Ding G Li X Hou X Zhou W Gong Y Liu F. REV-ERB in GABAergic neurons controls diurnal hepatic insulin sensitivity. Nature. (2021) 592:763–7. doi: 10.1038/s41586-021-03358-w PMC8085086 33762728

[59] Sulli G Rommel A Wang X Kolar MJ Puca F Saghatelian A. Pharmacological activation of REV-ERBs is lethal in cancer and oncogene-induced senescence. Nature. (2018) 553:351–5. doi: 10.1038/nature25170 PMC5924733 29320480

[60] Cho H Zhao X Hatori M Yu RT Barish GD Lam MT. Regulation of circadian behaviour and metabolism by REV-ERB-α and REV-ERB-β. Nature. (2012) 485:123–7. doi: 10.1038/nature11048 PMC3367514 22460952

[61] Liu H Zhu Y Gao Y Qi D Zhao L Zhao L. NR1D1 modulates synovial inflammation and bone destruction in rheumatoid arthritis. Cell Death Dis. (2020) 11:129. doi: 10.1038/s41419-020-2314-6 32071294 PMC7028921

[62] Berenbaum F Meng QJ. The brain-joint axis in osteoarthritis: nerves, circadian clocks and beyond. Nat Rev Rheumatol. (2016) 12:508–16. doi: 10.1038/nrrheum.2016.93 27305851

[63] Duffy T Bekki H Lotz MK. Genome-wide occupancy profiling reveals critical roles of foxO1 in regulating extracellular matrix and circadian rhythm genes in human chondrocytes. Arthritis Rheumatol. (2020) 72:1514–23. doi: 10.1002/art.41284 PMC7813518 32281255

[64] Dudek M Angelucci C Pathiranage D Wang P Mallikarjun V Lawless C. Circadian time series proteomics reveals daily dynamics in cartilage physiology. Osteoarthritis Cartilage. (2021) 29:739–49. doi: 10.1016/j.joca.2021.02.008 PMC8113022 33610821

[65] Akagi R Akatsu Y Fisch KM Alvarez-Garcia O Teramura T Muramatsu Y. Dysregulated circadian rhythm pathway in human osteoarthritis: NR1D1 and BMAL1 suppression alters TGF-β signaling in chondrocytes. Osteoarthritis Cartilage. (2017) 25:943–51. doi: 10.1016/j.joca.2016.11.007 PMC5438901 27884645

[66] Kantipudi S Jeckelmann JM Ucurum Z Bosshart PD Fotiadis D. The heavy chain 4F2hc modulates the substrate affinity and specificity of the light chains LAT1 and LAT2. Int J Mol Sci. (2020) 21:7573. doi: 10.3390/ijms21207573 33066406 PMC7589757

[67] Morio H Reien Y Hirayama Y Hashimoto H Anzai N. Protein kinase C activation upregulates human L-type amino acid transporter 2 function. J Physiol Sci. (2021) 71:11. doi: 10.1186/s12576-021-00795-0 33789576 PMC10716992

[68] Feng M Xiong G Cao Z Yang G Zheng S Qiu J. LAT2 regulates glutamine-dependent mTOR activation to promote glycolysis and chemoresistance in pancreatic cancer. J Exp Clin Cancer Res. (2018) 37:274. doi: 10.1186/s13046-018-0947-4 30419950 PMC6233565

[69] Xu J Jiang C Cai Y Guo Y Wang X Zhang J. Intervening upregulated SLC7A5 could mitigate inflammatory mediator by mTOR-P70S6K signal in rheumatoid arthritis synoviocytes. Arthritis Res Ther. (2020) 22:200. doi: 10.1186/s13075-020-02296-8 32867828 PMC7457370

[70] Zhao Y Xia Y Kuang G Cao J Shen F Zhu M. Cross-tissue analysis using machine learning to identify novel biomarkers for knee osteoarthritis. Comput Math Methods Med. (2022) 2022:9043300. doi: 10.1155/2022/9043300 35785145 PMC9246600

[71] Kaminski WE Wenzel JJ Piehler A Langmann T Schmitz G. ABCA6, a novel a subclass ABC transporter. Biochem Biophys Res Commun. (2001) 285:1295–301. doi: 10.1006/bbrc.2001.5326 11478798

[72] Petry F Ritz V Meineke C Middel P Kietzmann T Schmitz-Salue C. Subcellular localization of rat Abca5, a rat ATP-binding-cassette transporter expressed in Leydig cells, and characterization of its splice variant apparently encoding a half-transporter. Biochem J. (2006) 393:79–87. doi: 10.1042/bj20050808 16162093 PMC1383666

[73] He B Kang S Chen Z Liu X Wang J Li X. Hypercholesterolemia risk associated Abca6 does not regulate lipoprotein metabolism in mice or hamster. Biochim Biophys Acta Mol Cell Biol Lipids. (2021) 1866:159006. doi: 10.1016/j.bbalip.2021.159006 34274505

[74] Kubo Y Sekiya S Ohigashi M Takenaka C Tamura K Nada S. ABCA5 resides in lysosomes, and ABCA5 knockout mice develop lysosomal disease-like symptoms. Mol Cell Biol. (2005) 25:4138–49. doi: 10.1128/mcb.25.10.4138-4149.2005 PMC1087723 15870284

[75] DeStefano GM Kurban M Anyane-Yeboa K Dall’Armi C Di Paolo G Feenstra H. Mutations in the cholesterol transporter gene ABCA5 are associated with excessive hair overgrowth. PloS Genet. (2014) 10:e1004333. doi: 10.1371/journal.pgen.1004333 24831815 PMC4022463

[76] Palmer MA Dias IHK Smart E Benatzy Y Haslam IS. Cholesterol homeostasis in hair follicle keratinocytes is disrupted by impaired ABCA5 activity. Biochim Biophys Acta Mol Cell Biol Lipids. (2023) 1868:159361. doi: 10.1016/j.bbalip.2023.159361 37348644

[77] Fu Y Hsiao JH Paxinos G Halliday GM Kim WS. ABCA5 regulates amyloid-β peptide production and is associated with Alzheimer’s disease neuropathology. J Alzheimers Dis. (2015) 43:857–69. doi: 10.3233/jad-141320 25125465

[78] Pasello M Giudice AM Cristalli C Manara MC Mancarella C Parra A. ABCA6 affects the Malignancy of Ewing sarcoma cells via cholesterol-guided inhibition of the IGF1R/AKT/MDM2 axis. Cell Oncol (Dordr). (2022) 45:1237–51. doi: 10.1007/s13402-022-00713-5 PMC9747862 36149602

[79] van Leeuwen EM Karssen LC Deelen J Isaacs A Medina-Gomez C Mbarek H. Genome of The Netherlands population-specific imputations identify an ABCA6 variant associated with cholesterol levels. Nat Commun. (2015) 6:6065. doi: 10.1038/ncomms7065 25751400 PMC4366498

[80] Gai J Ji M Shi C Li W Chen S Wang Y. FoxO regulates expression of ABCA6, an intracellular ATP-binding-cassette transporter responsive to cholesterol. Int J Biochem Cell Biol. (2013) 45:2651–9. doi: 10.1016/j.biocel.2013.08.020 24028821

[81] Gkretsi V Simopoulou T Tsezou A. Lipid metabolism and osteoarthritis: lessons from atherosclerosis. Prog Lipid Res. (2011) 50:133–40. doi: 10.1016/j.plipres.2010.11.001 21115041

[82] Baudart P Louati K Marcelli C Berenbaum F Sellam J. Association between osteoarthritis and dyslipidaemia: a systematic literature review and meta-analysis. RMD Open. (2017) 3:e000442. doi: 10.1136/rmdopen-2017-000442 29435358 PMC5706481

[83] Choi WS Lee G Song WH Koh JT Yang J Kwak JS. The CH25H-CYP7B1-RORα axis of cholesterol metabolism regulates osteoarthritis. Nature. (2019) 566:254–8. doi: 10.1038/s41586-019-0920-1 30728500

[84] Cao C Shi Y Zhang X Li Q Zhang J Zhao F. Cholesterol-induced LRP3 downregulation promotes cartilage degeneration in osteoarthritis by targeting Syndecan-4. Nat Commun. (2022) 13:7139. doi: 10.1038/s41467-022-34830-4 36414669 PMC9681739

[85] Tsezou A Iliopoulos D Malizos KN Simopoulou T. Impaired expression of genes regulating cholesterol efflux in human osteoarthritic chondrocytes. J Orthop Res. (2010) 28:1033–9. doi: 10.1002/jor.21084 20108316

