# Supplementary material for: Identification of key biomarkers related to fibrocartilage chondrocytes for osteoarthritis based on bulk, single-cell transcriptomic data
Source: Front Immunol. 2024 Nov 21;15:1482361. doi: 10.3389/fimmu.2024.1482361 (PMC11617364; doi:10.3389/fimmu.2024.1482361)
Supplement: Supplementary file 1 [file DataSheet1.docx]

Supplementary Material

# Supplementary Figures and Tables


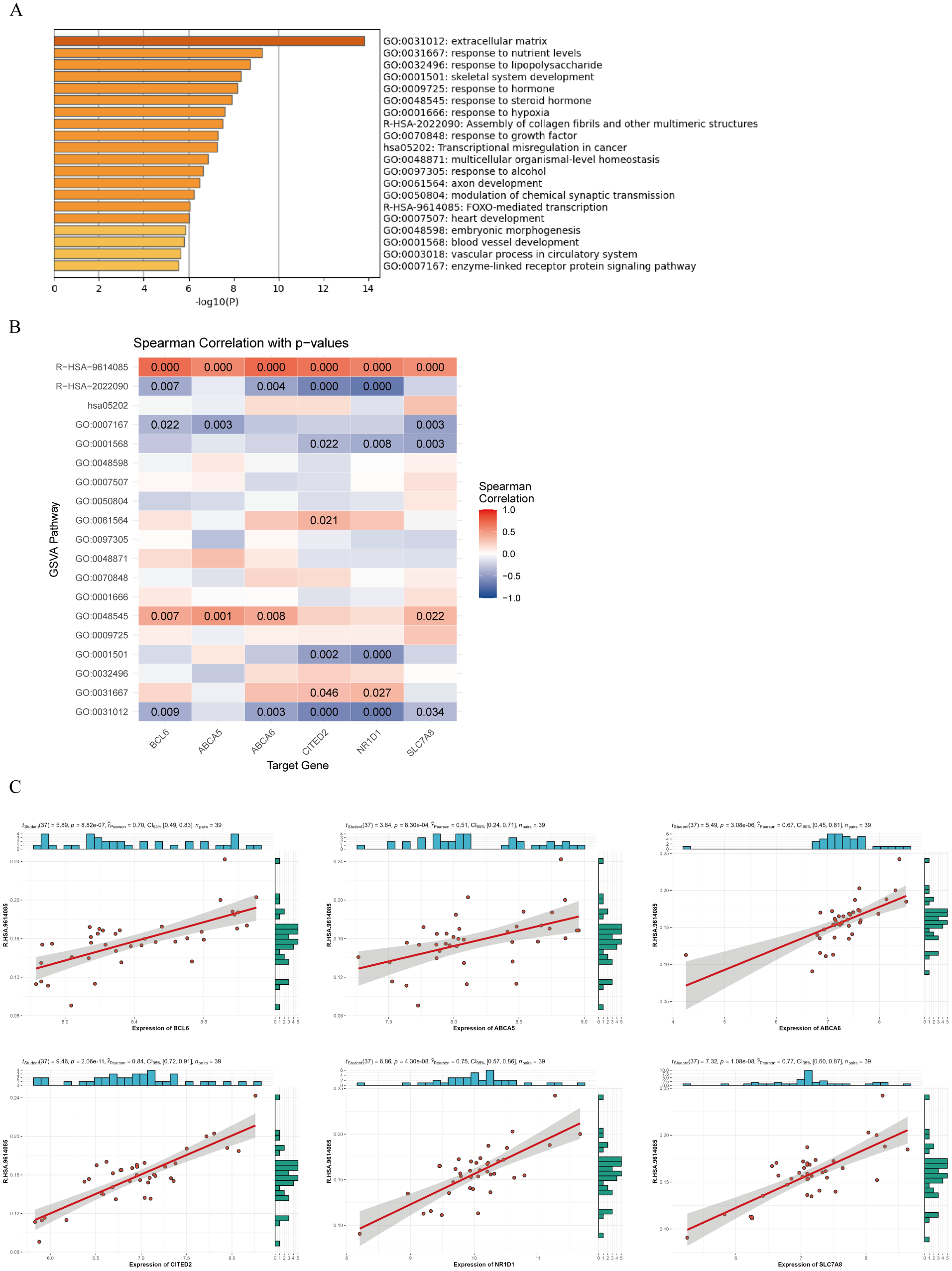


**Supplementary Figure 1.** Functional-enrichment analysis of DEGs and correlation analysis between the expression levels of the six biomarkers. **(A)** Bar graph of enriched terms across DEGs, colored by p-values. **(B)** Heatmap of correlations between GSVA pathways and target genes, with P-values annotated. **(C)** Scatter plot showing correlation between R-HSA-9614085 and the six genes.
